# Fall determinants in hospitalised older patients: a nested case control design - incidence, extrinsic and intrinsic risk in Malaysia

**DOI:** 10.1186/s12877-022-02846-6

**Published:** 2022-03-03

**Authors:** Fatt Soon Lee, Sondi Sararaks, Weng Keong Yau, Zen Yang Ang, Anis-Syakira Jailani, Zulkarnain Abd Karim, Lin Naing, Thillainathan Krishnan, Ai Reen Chu, Suria Junus, Mohd Shahril Ahmad, Norhayaty Sapiee, Vicneas Wary Veloo, Sangetavani Manoharan, Maimunah A. Hamid

**Affiliations:** 1 Department of Medicine, Kuala Lumpur Hospital, Jalan Pahang, 50586 Wilayah Persekutuan Kuala Lumpur, Malaysia; 2 Institute for Health Systems Research, National Institutes of Health, Ministry of Health Malaysia, ​Jalan Setia Murni U13/52, Seksyen U13 Setia Alam, 40170 Shah Alam, Selangor Malaysia; 3 Universiti Brunei Darussalam, Jalan Tungku Link, BE1410 Bandar Seri Begawan, Brunei; 4 Selayang Hospital, Lebuhraya Selayang-Kepong, 68100 Batu Caves, Selangor Malaysia; 5 Occupational Therapy Department, Tuanku Ja’afar Hospital, Jalan Rasah, 70300, Seremban, Negeri Sembilan Malaysia; 6 Occupational Therapy Unit, Kuala Lumpur Hospital, Jalan Pahang, 50586 Wilayah Persekutuan Kuala Lumpur, Malaysia; 7 Occupational Therapy Unit, Alor Gajah Hospital, Jalan Paya Datuk, 78000 Alor Gajah, Melaka Malaysia; 8 Office of the Deputy Director General of Health (Research & Technical Support), Ministry of Health Malaysia, Kompleks E, Presint 1, 62590 Putrajaya, Malaysia

**Keywords:** Elderly, Falls, Inpatient, Hospitals, Determinants, Risk, Intrinsic, Extrinsic

## Abstract

**Background:**

The older person is at greater risk of falls due to multiple intrinsic and extrinsic factors. This is compounded when the elderly is admitted to hospitals, as they are acutely ill and placed in an unfamiliar environment. Delirium and polypharmacy further complicate these problems. As falls reflect quality of care with potential for grave outcomes, this study aimed to identify the extent and risk of falls in public hospitals.

**Methods:**

We conducted a nested case control study in 12 public hospitals in Malaysia. In the cohort section, we screened all inpatients 60 years of age and above daily until discharge, or the end of the study period. Daily, we identified those who fell, inclusive of near falls, in the preceding 24 h. Our enumerators interviewed patients on experience of fall, and supplemented data from the nurses and caregivers. For each case, ten controls were chosen.

**Results:**

The incidence of falls/near falls was 1.0 per 1000 patient days (95% CI: 0.9, 1.1). Intrinsic risk factors found to be significant included patients who were not from a nursing home or not cared for by a domestic helper prior to admission, had prior history of indoor fall either in home or hospital, had four or more clinical diagnoses or exited from the bed on the weak side. Significant extrinsic factors were the absence of transfer bar in toilet, call bells, light switches or walking aids that were not within reach, as well as not having a walking aid. Non-sturdy chair was associated with lesser falls than when sturdy chairs with armrests were present.

**Conclusion:**

Querying patients for falls produced better results than incident reporting. Several intrinsic factors such as history of indoor or in-hospital fall, having four or more clinical diagnoses or exiting from weaker side and residence history may help to identify those at higher risk. Addressing significant extrinsic factors such as transfer bars and the identification of switches may help in reducing falls risk in hospitals.

**Trial registration:**

This study was registered in National Medical Research Register of Malaysia (NMRR-07-772-1044; date 26/05/2008) with Ethics Approval from Medical Research and Ethics Committee (MREC: MRG-07-LOI-HSR-1).

## Background

Falls are usually not within the attention of healthcare workers; instead, they are dismissed as part of ageing. In reality, published figures quote a prevalence of 28-35% in the community [1]. This risk increases when the person is elderly, unwell and admitted into an unfamiliar environment such as a hospital.

Falls are not innocuous. Ten percent of falls lead to injuries; this ranges from soft tissue contusion to fractures. In addition, falls may result in psychological trauma and functional impairment in older patients leading to immobility and functional dependence [2]. This increases the risk of nursing home placement [3, 4].

Falls in the older person commonly result from interactions between intrinsic and extrinsic (environmental) factors [5]. The elderly patient may have comorbidities such as poor vision, arthritis, and previous stroke, as well as be on polypharmacy. Acute illnesses such as confusion due to delirium, contribute to intrinsic factors for falls [5, 6]. Hence, falls may be a marker of an acute illness or an exacerbation of a chronic illness [7]. These interactions become more complex as the number of comorbidities increases with advancing age. In addition, extrinsic factors such as lighting, flooring and clutter can increase the risk [8].

In acute care hospitals, the incidence of reported falls varies from 0.6 to13 falls per 1000 patient days [9, 10]. Facilities dealing with confused, frail patients and active rehabilitation would logically have a higher incidence of falls.

Traditionally, data on hospital falls are from incident reporting but there are reservations on this mechanism’s ability to portray the true rate due to underreporting, as underreporting could be as large as 50% [11, 12]. Agents influencing incident reporting include time pressures, “blame culture”, perceived medico-legal liability, and perception of staff on whether their report would improve patient safety [11, 13, 14]. One of the ways suggested to improve reporting includes patient self-reporting of these adverse events [15].

The true incidence and risk factors of hospital falls in the country are not known, although in Malaysia’s public hospitals, falls formed the third highest reported incident for the year 2006-2008 [16]. Given the degree of under-reporting in incident reports [12], the paucity of data on magnitude of the problem in public hospitals in Malaysia as well as the possible grave outcomes resulting from falls that are potentially avoidable, which subsequently reflects the quality of care, [13, 17] we decided to look into the incidence, fall events and its risk factors among hospitalised elderly. This could aid in efforts to anticipate and prevent falls in hospitalised elderly.

## Methods

Generally, Malaysia has a two-tier system where the healthcare system consists of public and private sectors [18]. The public hospital system, which is managed by Ministry of Health (MOH) Malaysia, consists of state-level, major specialist, minor specialist and district hospitals. A state hospital provides up to 45 specialty and subspecialty services, while major specialist hospitals deliver up to 20 specialty services [19]. Wards in public hospitals are generally divided into three classes (1st class, 2nd class and 3rd class, mainly based on room costs) and they are further divided into male and female adult wards [20]. In practice, 3rd class wards receives the majority of acute cases and constitutes 86.4% of total beds in public hospitals, with only 0.6% of total beds in 1st class wards [21]. The beds inclusive of the services provided in the third class ward are heavily subsidised (98% subsidy) by the government where occupants pay a minimal amount of MYR3 for daily general ward charges [21–23]. In addition, there is an avenue to waive the charges if the occupants who are in dire need [24]. In contrast, the charges in 1st and 2nd class wards are higher and either borne by the patient or his/her employers if he/she so wishes to utilise the facilities [21]. The range of services provided to all groups of patients are similar and from the same respective departments [22] (Appendix 1). In 2015, the official bed capacity in MOH hospitals was 41,389 beds with average bed occupancy rate of 71.09% and a mean length of stay of 4.1 days [25]. In the same year, these public hospitals received 2,526,205 admissions (paid and free of charge), while paid admissions to 3rd class ward alone were estimated to be 1,968,000, indicating that the majority of admissions in public hospital were third class admissions [21, 25] (Appendix 1).

### Overview of study design

In the cohort segment (Part I), all inpatients 60 years and above in general wards were interviewed on the first day of admission or at the start of the study, and followed up daily until discharge, or the end of study period. Enumerators interviewed all these patients daily to ask if they had fallen in the preceding 24 h. In the nested case control portion (Part II), carried out concurrently with Part I, patients who had fallen in the preceding 24 h were interviewed for additional information. For each case, ten controls were chosen. In this study, falls defined as ‘any event when the participant unexpectedly came to rest on the ground, floor or another lower level’, and includes a near fall, defined as ‘the person seems to fall, but could prevent the fall by catching or leaning on a person or a thing (e.g., a chair, a drawer or a table) [26, 27]. Excluded were inpatients in intensive care or high dependency wards. Patients fully or partially bed-bound and admitted into general wards were not excluded.

### Sampling methods and sample size

Hospitals were selected from the 120 public hospitals listed in the MOH Information and Documentation System (IDS) in 2006. A total of twelve (12) hospitals, consisting of state hospitals, hospitals with specialists and hospitals without specialists located in East and West Malaysia, with high admission rates of elderly were selected using simple random sampling (SRS) without replacement method. We conducted a nested case control study from May to November 2007.

Using EpiCalc 2000 [28], estimating a proportion of 0.6% of falls (expected) with a precision of + 0.2% at 95% confidence level, a minimum sample size of 5727 was required for the cohort (Part I). For Part II, we used the following parameters for sample size calculation: a ratio of 1:10 case to control, an Odds Ratio (OR) to be detected of 2.5, a significance level of 0.05, power of 80%, and an estimate of 30% of the community with history of previous falls [6, 29]. EpiCalc 2000 calculates a minimum sample of 41 cases and 410 controls for this study.

### Data collection process

Training session on the protocol, illustrated manual and instruments was done centrally for all enumerators and supervisors from participating hospitals before the study commenced. Participating hospitals appointed nurses as study supervisors and several authors trained them on the protocol, quality control and required monitoring of enumerator performance during data collection.

#### Part I- screening for falls – cohort

Part I comprised of screening of all patients 60 years and above admitted to the wards in the participating hospitals. At the first interview (of patient or caregiver), we captured information on socio-demographic characteristics, history of fall in the last 24 h (during admission), history of previous falls (before admission) and other risk factors. We obtained disease characteristics such as clinical diagnosis and medication information from medical records. To identify those who fell in the preceding 24 h, enumerators enquired daily on experience of fall from the nurses in the ward and every patient or caregiver. Patients who had a fall were recruited for Part II.

#### Part II-nested-case control

For each fall identified during the study period in Part I, we chose 10 controls, aged ≥60 years, matched by ward type (1st, 2nd, 3rd class) and discipline (medical, surgical, orthopaedics, and radiotherapy/oncology). We chose controls based on physical proximity to the patient’s bed, and from the same ward, as most wards had acute, subacute and convalescent sections for stable patients. If there were insufficient numbers for controls from within the same ward, we extended the search to other wards of the same discipline. The search was extended to wards of different disciplines only when there were not enough patients for controls. Enumerators captured data on falls epidemiology (when, where and activity just prior to the fall), site and severity of injury, intrinsic risk factors (medications, blood pressure, functional status) and extrinsic risk factors (bed area, bathroom, other areas) in face-to-face interviews with either the subjects, nurse in the ward or their caregivers, as well as from medical records.

### Instruments

Instruments used in the study included Falls Screening Forms (I and II) for intrinsic and extrinsic factors and Barthel Index, an instrument to assess the capacity of the respondent to perform activities of daily living [30]. Form I covered areas such as socio-demography, medical, and previous falls history, while Form II captured intrinsic and extrinsic factors of the current fall experience.

### Ethical considerations

The study protocol was approved by the Medical Research and Ethics Committee, Ministry of Health Malaysia (MRG-07-LOI-HSR-1) with the study registration number NMRR-07-772-1044, dated 26/05/2008. During the study period, the enumerators notified all fall events detected to the ward supervisor or senior nurse in charge to enable the appropriate measures taken as per hospital standard operating procedure.

### Statistical analysis

The data was manually entered using EpiInfo 2000. A second research assistant verified 10 % of randomly selected data (constituting approximately 1400 records) for data entry errors [31]. A more detailed check was not done as we did not detect substantial errors. However, for key variables, 100% verification was carried out. After data entry verification, we explored numerical variables, including calculating mean and SD, median and IQR, minimum and maximum, and plotted histograms. We explored variables of diagnosis, comorbidities and drugs administered, and identified and reclassified categories with small numbers.

STATA version 8.0 [32] was used for all statistical analyses. To calculate incidence of falls, the number of patients who fell per 1000 admissions was calculated using estimation of proportions. We used Poisson estimation method to calculate falls per 1000 patient-days.

Using survey data analysis commands in STATA with primary sampling unit, finite population correction, and probability weights, we applied conditional logistic regression to identify the contributory factors for falls. We used univariable conditional logistic regression analyses for each independent variable and backward variable selection method to obtain the preliminary main effect model (PMEM). We obtained six potential PMEMs and selected the best PMEM considering biological plausibility.

We checked the selected PMEM model for all possible two-way interactions for significance and multi-collinearity by running the model with linear regression command (“regress”) followed by obtaining the variance-inflation-factor. Basic model fit assessment was done using predicted probability and we interpreted the results using this final model.

## Results

### Sample description

#### Part I: cohort

14,108 patients were recruited in Part I. Slightly more than half were males (54.0%) and Malays accounted for 47.9% of the sample, with the largest age group between 65 to 74 years (64.6%). Comparison of the sample characteristics with the population admitted to MOH hospitals in 2007 (Table 1) showed those 85 years and above and ethnic groups were under-represented. Of the 14,108, we detected 82 falls among 81 patients; one subject fell twice during the hospital stay.

**Table 1:** Comparison of characteristics of respondents in cohort (Part I) with MOH hospital admission for year of study

Socio Demographic	Cohort (Part I)		MOH hospital admission for Year of Study^**c**^		***P*** value*
	Count	%	Count	%	
Overall	14,108	100.0	385,264	100.0	–
^a^Age category (years old) (> = 65: *n* = 10,413)					
55 – 64	3678	–	162,883	–	–
65 – 74	6985	64.6	143,165	64.4	0.632
75 – 84	2917	29.8	65,411	29.4	0.310
> =85	511	5.6	13,805	6.2	0.004
Missing / Unknown	17	–	0	–	–
Sex					
Male	7769	54.0	207,759	53.9	0.822
Female	6339	46.0	177,505	46.1	0.822
Ethnic group					
Malay	6370	47.9	205,875	53.4	< 0.001
Chinese	4356	26.3	98,032	25.4	0.016
Indian	1699	12.6	39,467	10.2	< 0.001
Other indigenous	1498	12.0	34,499	9.0	< 0.001
Others	185	1.2	7391	1.9	< 0.001
Length of stay (LOS, days)	14,108	6.6	385,264	4.4^b^	–
Ward Class					
1st class	1104	7.8	–	–	–
2nd class	835	5.9	–	–	–
3rd class	12,169	86.3	–	–	–
Discipline					
Medical	9504	67.4	–	–	–
Surgical	2330	16.5	–	–	–
Orthopaedic	1184	8.4	–	–	–
Radiotherapy/Oncology	1090	7.7	–	–	–
Previous Fall					
Yes	10,954	77.6	–	–	–
No	3154	22.4	–	–	–
Barthel Index					
0-5	207	1.5	–	–	–
6-10	98	0.7	–	–	–
11-15	168	1.2	–	–	–
16-20	409	2.9	–	–	–
Missing / Unknown	13,226	93.8	–	–	–

Note:

^a^ For age, comparison was made between ages 65 and above as no data was available for the age group of 60-64 years in MOH Hospital admissions

* *P* value was calculated using EpiCalc 2000, using comparing two proportions (percentages)

^b^ Average length of stay (all age groups)

^c^ Data refer to MOH admissions for those age 55 years old & above, except for LOS

The incidence of falls was 1.0 per 1000 patient days (95%CI: 0.9, 1.1) (Table 2). Medical department patients had higher incidence than orthopaedic, radiotherapy/oncology or surgical disciplines; older ages (71-75 years) had higher incidence than those who aged 60-65 years. In addition, those with length of stay in hospital (LOS) 4 days or less had higher incidence (1.6/1000 patient days; 95%CI: 1.3, 1.9) than those with LOS 20 - < 25 days (0.4/1000 patient days; 95%CI: 0.2, 0.7) (Table 2).

**Table 2:** Incidence of fall or near fall per 1000 patient days (Part I)

Characteristics	Sample size	Count	Per 1000 patient days	95% confidence interval
Overall	14,108	82	1.0	0.9-1.1
Age category (years old)				
60 – 65	4448	24	0.9	0.7-1.1
66 – 70	3784	19	1.0	0.8-1.2
71 – 75	2880	18	1.4	1.2-1.8
76 – 80	1736	9	0.5	0.3-0.7
> 80	1243	12	1.3	0.9-1.7
Missing / Unknown	17	0	–	–
Sex				
Male	7769	41	0.9	0.7-1.0
Female	6339	41	1.2	1.1-1.4
Ethnic group				
Malay	6370	41	1.4	1.2-1.6
Chinese	4356	26	0.7	0.6-0.9
Indian	1699	12	1.0	0.7-1.3
Other indigenous	1498	3	0.4	0.2-0.6
Others	185	0	–	–
Length of stay (LOS, days)				
0	267	0	–	–
1 – 4	7075	21	1.6	1.3-1.9
5 – 9	3780	16	1.2	1.0-1.5
10 - < 15	1366	13	0.6	0.4-0.9
15 - < 20	615	10	1.5	1.1-2.0
20 - < 25	323	3	0.4	0.2-0.7
> =25	602	19	0.6	0.4-0.8
Missing	80	0	–	–
Ward Class				
1st class	1104	10	1.0	0.6-1.5
2nd class	835	3	0.3	0.1-0.7
3rd class	12,169	69	1.1	1.0-1.2
Discipline				
Medical	9504	63	1.3	1.1-1.4
Surgical	2330	6	0.4	0.2-0.6
Orthopaedic	1184	8	0.8	0.5-1.1
Radiotherapy/Oncology	1090	5	0.4	0.2-0.7
Previous Fall				
Yes	3154	43	1.9	1.6-2.2
No	11,039	39	0.6	0.5-0.7
Barthel Index				
0-5	207	2	1.0	0.3-2.3
6-10	98	1	4.7	3.0-7.1
11-15	168	2	0.8	0.2-2.3
16-20	409	3	0.8	0.3-1.6
Missing / Unknown	13,226	74	1.0	0.9-1.1

Note: Analysis done using Poisson Distribution in STATA 8

#### Part II: nested case control

There were slightly more males (58.9%) and Malays (52.7%), with the commonest age group 65 to 74 years (62.6%). Female admissions were under-represented (Table 3).

**Table 3:** Comparison of characteristics of respondents in nested case control (Part II) with MOH hospital admissions for year of study

Socio Demographic	Nested case control (Part II)		MOH hospital admission for Year of Study^**c**^		***P*** value*
	Count	%	Count	%	
Overall	882	100.0	385,264	100.0	–
^a^Age category (years old) (> = 65: *n* = 660)					
55 – 64	222	–	162,883	–	–
65 – 74	410	62.6	143,165	64.4	0.242
75 – 84	207	30.4	65,411	29.4	0.291
> =85	43	6.9	13,805	6.2	0.806
Sex					
Male	506	58.9	207,759	53.9	0.044
Female	376	41.1	177,505	46.1	0.044
Ethnic group					
Malay	440	52.7	205,875	53.4	0.038
Chinese	249	27.1	98,032	25.4	0.063
Indian	128	12.6	39,467	10.2	< 0.001
Other indigenous	46	5.4	34,499	9.0	< 0.001
Others	19	2.2	7391	1.9	0.699
Length of stay (LOS, days)	882	15.1	385,264	4.4^b^	–
Ward Class					
1st class	84	9.5	–	–	–
2nd class	38	4.8	–	–	–
3rd class	760	85.7	–	–	–
Discipline					
Medical	624	70.0	–	–	–
Surgical	96	13.2	–	–	–
Orthopaedic	116	12.7	–	–	–
Radiotherapy/Oncology	46	4.2	–	–	–
Previous Fall					
Yes	601	66.6	–	–	–
No	281	33.4	–	–	–
Barthel Index					
0-5	216	26.0	–	–	–
6-10	54	5.7	–	–	–
11-15	135	15.4	–	–	–
16-20	477	52.8	–	–	–

Note:

^a^For age, comparison was made between ages 65 and above as no detailed data available for the age group of 60-64 years in MOH Hospital admissions date

* *P* value was calculated using EpiCalc 2000, using comparing two proportions (percentages)

^b^Average length of stay in hospital (all age groups)

^c^All data refer to MOH admissions for these age 55 years old & above, except for LOS

### Risk factors for falls

#### Univariable model

##### Intrinsic factors

In the univariable model, patients whose LOS were 2-3 days and 8-12 days were four times (OR = 4.00; 95% CI: 1.30, 12.30; *p* = 0.021) and three times (OR = 2.85; 95% CI: 1.16, 7.01; *p* = 0.027) more likely, respectively, to fall compared to those who stayed for a day. Patients with Barthel Index of 5 to 9 (OR = 2.47; 95% CI: 1.28, 4.77; *p* = 0.012) and of 10 to 14 (OR = 2.38; 95% CI: 1.13, 5.00; *p* = 0.027) were twice more likely to fall compared to patients with Barthel Index 20 (Table 4). Similarly, patients whose walking aid was not within reach or was without a walking aid were twice more likely to fall compared to those with a walking aid that was within reach (OR = 1.94; 95% CI: 1.17, 3.22; *p* = 0.016). Patients who had a prior history of a fall (once) were twice more likely to fall compared to those who never fell before (OR = 2.47; 95% CI: 1.09, 5.63; *p* = 0.034).

**Table 4:** Crude and adjusted effect of predictors for inpatient fall

Characteristics		Crude Effect				Adjusted Effect			
		OR	95% Confidence Intervals		*p* value	OR	95% Confidence Intervals		*p* value
	n		Lower	Upper			Lower	Upper	
**A. Socio demographics**									
Age category (years old) (R:60-64)	222								
65-69	220	1.52	0.72	3.20	0.238	1.67	0.63	4.42	0.267
70-74	190	1.17	0.61	2.24	0.605	1.05	0.51	2.16	0.886
75-79	130	1.36	0.52	3.61	0.489	1.83	0.47	7.12	0.342
80+	120	1.45	0.68	3.06	0.293	1.26	0.57	2.81	0.526
Sex (R: Male)	506								
Female	376	1.15	0.59	2.25	0.643	0.76	0.38	1.51	0.387
Discipline (R: Medicine)	602								
Surgery	118	0.87	0.19	4.03	0.847	﻿–	﻿–	﻿–	﻿–
Orthopaedic	118	0.47	0.15	1.49	0.174	﻿–	﻿–	﻿–	﻿–
Radiotherapy/Oncology	44	1	†	†	†	﻿–	﻿–	﻿–	﻿–
Stay with whom (R: Alone, Spouse only or Children only)	561								
Domestic helper, Nursing Home or Combination	321	0.71	0.48	1.04	0.070	0.61	0.41	0.90	0.019
**B. Intrinsic Factors**									
Length of stay (LOS, days) (R: 1)	23								
2-3	105	4.00	1.30	12.30	0.021	4.81	0.70	32.83	0.098
4-7	226	1.89	0.37	9.62	0.401	2.64	0.23	29.92	0.388
8-12	173	2.85	1.16	7.01	0.027	4.71	0.69	32.31	0.102
13+	355	3.02	0.62	14.64	0.147	3.78	0.58	24.79	0.144
Barthel Index (R: 20)	227								
0-4	187	1.35	0.60	3.03	0.421	0.70	0.28	1.75	0.407
5-9	83	2.47	1.28	4.77	0.012	1.85	0.79	4.35	0.138
10-14	173	2.38	1.13	5.00	0.027	1.53	0.82	2.86	0.155
15-18	161	1.59	0.64	3.93	0.277	1.26	0.53	3.00	0.560
19	51	2.14	0.57	8.11	0.228	1.37	0.33	5.78	0.631
Walking aid (R: Within reach)	257								
Don’t Have/Not within reach	625	1.94	1.17	3.22	0.016	2.45	0.99	6.04	0.052
No. of diagnosis (R: 0-1)	569								
2-3	225	2.13	0.45	10.09	0.299	2.42	0.61	9.65	0.182
4-8	88	3.47	1.00	12.04	0.05	3.50	1.11	11.01	0.035
No. of medication (R: 0-3)	290								
4-9	565	0.77	0.46	1.29	0.283	0.67	0.44	1.03	0.063
10+	27	1.21	0.44	3.31	0.685	0.97	0.43	2.16	0.929
Exit bed towards (R: Strong side / No weakness)	657								
Weak side	152	2.56	0.85	7.74	0.086	3.76	1.21	11.72	0.027
Combination of strong side and weak side	73	0.19	0.01	2.50	0.177	0.12	0.01	1.67	0.103
Impaired Far vision (R: None/Mild)	700								
Severe	182	1.00	0.72	1.39	0.982	0.73	0.49	1.09	0.105
**C. Extrinsic Factors**									
Bathroom condition (R: Dry)	608								
Wet	274	3.72	1.33	10.44	0.018	﻿–	﻿–	﻿–	﻿–
Call bells/Light (R: Within reach)	241								
Not present	495	0.83	0.42	1.61	0.535	1.26	0.56	2.81	0.532
Not within reach	134	2.13	1.08	4.23	0.034	4.04	1.33	12.26	0.019
Missing/Unknown^a^	12	﻿–	﻿–	﻿–	﻿–	﻿–	﻿–	﻿–	﻿–
Toilet transfer bar installed (R: No)	224								
Yes	658	0.54	0.33	0.89	0.021	0.50	0.27	0.94	0.034
Exit doors in bathroom (R: No signage)	113								
Letter only	326	0.08	0.03	0.21	< 0.001	﻿–	﻿–	﻿–	﻿–
Picture only	113	2.01	0.32	12.78	0.414	﻿–	﻿–	﻿–	﻿–
Letter & Picture	267	0.61	0.22	1.68	0.301	﻿–	﻿–	﻿–	﻿–
Missing/Unknown	63	0.11	0.03	0.48	0.008	﻿–	﻿–	﻿–	﻿–
Chair (R: Sturdy with arm rest)	121								
Not sturdy	525	0.29	0.09	0.89	0.034	0.22	0.07	0.67	0.013
Sturdy without arm rest	176	0.55	0.30	1.01	0.054	0.70	0.19	2.59	0.55
No chair	60	0.69	0.16	3.06	0.589	0.44	0.14	1.39	0.14
Bed rail (R: Both raised)	200								
Both lowered	343	1.43	0.64	3.19	0.335	﻿–	﻿–	﻿–	﻿–
Only one side lowered	112	0.29	0.11	0.77	0.019	﻿–	﻿–	﻿–	﻿–
No bed rail or not functioning	227	0.41	0.15	1.14	0.079	﻿–	﻿–	﻿–	﻿–
Non-slip mat (R: Having a mat)	30								
No mat	852	1.04	0.19	5.62	0.955	﻿–	﻿–	﻿–	﻿–
**D. Fall history**									
Previous fall (R: No)	601								
Yes	281	2.21	0.93	5.24	0.067	﻿–	﻿–	﻿–	﻿–
Number of previous falls (R: No falls)	601								
Once	154	2.47	1.09	5.63	0.034	﻿–	﻿–	﻿–	﻿–
Twice	58	2.64	0.87	8.00	0.079	﻿–	﻿–	﻿–	﻿–
Thrice or more	66	1.27	0.20	8.02	0.779	﻿–	﻿–	﻿–	﻿–
Missing/Unknown^a^	3	﻿–	﻿–	﻿–	﻿–	﻿–	﻿–	﻿–	﻿–
Most recent fall (R: < 6 months)	137								
> =6 months	141	2.17	0.67	6.98	0.169	﻿–	﻿–	﻿–	﻿–
No falls	601	0.72	0.22	2.37	0.550	﻿–	﻿–	﻿–	﻿–
Missing/Unknown^a^	3	﻿–	﻿–	﻿–	﻿–	﻿–	﻿–	﻿–	﻿–
Place of previous falls (R: Never fall)	601								
Out of Home	111	1.26	0.58	2.75	0.515	1.59	0.75	3.38	0.197
Home/Hospital/Combine	165	2.72	0.99	7.47	0.052	3.08	1.13	8.38	0.032
Missing/Unknown^a^	5	﻿–	﻿–	﻿–	﻿–	﻿–	﻿–	﻿–	﻿–
Presence of carer at time of fall (R: None)	476								
Staff/Relative	406	0.86	0.40	1.85	0.668	﻿–	﻿–	﻿–	﻿–
Activity before previous falls (R: No fall)	601								
Transfer from bed	20	5.48	0.59	50.95	0.118	﻿–	﻿–	﻿–	﻿–
Toilet (passed urine/bowels open)	27	1.72	0.42	6.99	0.407	﻿–	﻿–	﻿–	﻿–
Bathing	27	5.70	1.46	22.21	0.018	﻿–	﻿–	﻿–	﻿–
Walking	146	2.11	0.73	6.08	0.146	﻿–	﻿–	﻿–	﻿–
Others	61	0.91	0.18	4.50	0.893	﻿–	﻿–	﻿–	﻿–

† cannot obtain statistics due to small cell

^a^ omitted from conditional logistic regression analysis

##### Extrinsic factors

Wet bathroom conditions were four times more likely to cause a fall (OR = 3.72; 95% CI: 1.33, 10.44; *p* = 0.018). Likewise, bathing activity was six times more likely to cause falls (OR = 5.70; 95% CI: 1.46, 22.21; *p* = 0.018). Patients whose call bells or light switches not within reach were twice more likely to fall compared to those whose call bells or light switches were within reach (OR = 2.13; 95% CI: 1.08, 4.23; *p* = 0.034) (Table 4).

Patient in wards with toilet transfer bar installed (OR = 0.54; 95% CI: 0.33, 0.89; *p* = 0.021) and patients in wards with letter-based signage for bathroom exit doors only (OR = 0.08, 95% CI: 0.03, 0.21; *p* < 0.001) were less likely to fall compared to those in wards without toilet transfer bar installed and those in wards without a signage, respectively. Interestingly, patients whose bedside chair was not sturdy were less likely to fall compared to those whose chair was sturdy and with armrest (OR = 0.29; 95% CI: 0.09, 0.89; *p* = 0.034). Moreover, beds with only one side of rails lowered were found to have protective effect from falling (OR = 0.29; 95% CI: 0.11, 0.77; *p* = 0.019).

#### Adjusted model

In the multivariable analysis, age, sex, staying with whom before current admission, place of previous falls, Barthel Index, installation of toilet transfer bar, usage of walking aid, having call bells in the ward, sturdiness of chair, number of diagnoses, length of stay, number of medications, impaired far vision, and exiting from bed on the weaker side were included in the final model.

After controlling for other variables in the model, patients who stayed with a domestic helper, in a nursing home or a combination of situations before the current admission were less likely to fall compared to those who lived alone or stayed with spouse or children only (OR = 0.61; 95%CI: 0.41, 0.90; *p* = 0.019) (Table 4). Patients with previous fall at home and / or in hospital were 3 times more likely to fall compared to those did not fall before (OR = 3.08; 95%CI: 1.13, 8.38; *p* = 0.032).

Those with 4 to 8 diagnoses were approximately 3.5 times more likely to fall compared to those with single or no diagnosis (OR = 3.50; 95%CI: 1.11, 11.01; *p* = 0.035). Patients who exited from bed on the weaker side (OR = 3.76; 95%CI: 1.21, 11.72; *p* = 0.027) and patients in wards with call bells or light not within reach (OR = 4.04; 95%CI: 1.33, 12.26; *p* = 0.019) were 4 more times likely to fall compared to those with no weakness and those with call bells or light within reach, respectively.

On the other hand, patients in wards with a toilet transfer bar installed were less likely to fall compared with patients in wards without a transfer bar (OR = 0.50; 95%CI: 0.27, 0.94; *p* = 0.034). Intriguingly, having a non-sturdy chair near was found to be protective against fall compared to a sturdy chair with armrest (OR = 0.22; 95%CI: 0.07, 0.67; *p* = 0.013).

## Discussion

The incidence of falls was 1.0 per 1000 patient days, with a longer length of stay greater than 20 days associated with a lower incidence. Intrinsic risk factors found to be significant included prior history of indoor or in hospital fall, walking aids that were not within reach, or not having a walking aid and exiting from the bed on the weaker side. In contrast, living in a nursing home or cared for by a domestic helper prior to admission was a protective factor for elderly inpatient falls. Significant extrinsic factors were the absence of transfer bar in toilet and call bells/light switches.

### Incidence

Compared to rate of fall among elderly inpatients in Italian and German acute care hospitals which ranged from 4 to 6 falls per 1000 patient days [33, 34], the rate of fall of elderly inpatients in MOH Malaysia hospitals (1.0 per 1000 patient days) was relatively low. Nevertheless, our study detected more falls than the hospital Incident Reporting process [35]; the latter captured only approximately half of falls that had occurred, yielding a 50% under-reporting rate [35]. The issue of under-reporting was also described by several studies [11, 36, 37] and Waring [14] postulated that the under-reporting might be due to “rejection of excessive administrative duties”, perceived as unavoidable and not within control, and therefore meaningless to report while Evans et al [37]. noted that senior doctors were less likely to report incidents as compared to junior doctors.

### Intrinsic factors

We found that inpatients with higher number of comorbidities (i.e., 4 to 8 diagnoses) had higher risk of fall as compared those one or no comorbidity. This is consistent with the findings from several studies which indicated that risk of fall is higher in those community-dwelling older adults with higher number of comorbidities [38–40]. Nevertheless, we could not further categorise the diagnoses due to varied response and small sample size. On the other hand, our study did not reveal any differences across age or gender. The functional status, as measured by Barthel index, did not prove to be significant. Patients who transferred on their weaker side instead was a crucial factor.

Polypharmacy has frequently been associated with falls [34, 41]. However, in our study, patients on four or more medications were at lower risk of falls (approaching marginal significance, *p* = 0.064). This could be because the study hospitals are mainly for acute care. Hospitalised patients treated with more medications could indicate higher illness severity, therefore there could be less falls because of increased attention by amongst medical staff or the ill condition itself led to decreased activity leading to less risk of falls. We could not analyse for presence of specific drug categories such as those likely to predispose to falls, e.g., sedatives, due to insufficient sample size for the different medication types amongst fallers.

Consistent with previous studies [42–45], previous falls, either in homes or hospitals, significantly increased the likelihood of elderly inpatient fall. Furthermore, exiting from the bed on the weaker side, i.e., a motor deficit, significantly increased the likelihood of fall [46]. We found that patients who were from nursing homes or taken care of by a domestic helper prior to admission were found to have lesser tendency for falls when hospitalised. In contrast, elderly patients in institutionalised care (i.e., nursing home) (32.8%) reported more falls than community dwellers (18.9%) [47, 48]. Meanwhile, Romli et al. [49] found no significant difference in risk of fall between community dwellers with or without domestic helper. We postulate that this difference could be because the frailer population was admitted for an acute illness, rendering them more dependent thus leading to lower fall rate. Although far vision was not found to be a determinant of falls in this study, poor vision has been correlated [50] with risk of falls and fractures [51]. Multifocal glasses have disadvantages because the lower lenses blur floor-level objects at critical distances for detecting environmental hazards [52]. This factor may represent a significant problem for older people, as they are more likely to fall over a hazardous object.

### Extrinsic factors

Out-of-reach switches as well as absent transfer bars were associated with increased falls, as identified by Carter et al. [53] One unexpected finding was that the presence of non-sturdy chair contributed to lower risk of falls. This is in contrast with other studies [53–56]. A possible reason for this finding is that non-sturdy chairs may contribute to patients feeling unsafe to use these chairs as support, and hence might not ambulate.

### Possible implications for clinicians or policymakers

The study suggests that patient reports represent a valuable source of events inside the hospital, as noted elsewhere [15] and active enquiry method, as part of a daily assessment to detect fall experience, is an action recognised and recommended for the elderly [5]. For better targeting of patients, this method may be used on patients with risk factors, as risk of falls increases with multiple factors [57].

Currently, the Morse Fall Scale is used to assess risk of falls for all inpatients in MOH hospitals during admission [58]. With early detection and practical interventions, one could reduce this risk of fall in high-risk inpatients. For instance, healthcare workers could be trained to ensure items such as call bells are within reach and to educate inpatients with a high risk of fall not to exit on the weaker side. Moreover, this study found that conducive environment is crucial to reduce the risk of fall. As elderly with functional impairment, acute illness or previous falls generally have a higher risk of fall, they can be greatly assisted by modifying the environment, such as installing transfer bars or grab bars, or keeping floors dry. Although our findings are affected by lack of details on several intrinsic factors known to affect falls such as illness types, co-morbidities, cognitive function, frailty level as well as medications [38, 39, 44, 45, 59–62], interventions on extrinsic factors are still valuable towards the creation of a safer environment, irrespective of individual functioning. Future studies could explore the impact of extrinsic interventions in reducing inpatient falls.

### Strengths, weaknesses and future research

We screened more than 14,000 patients across 12 acute care public hospitals and collected controls in the ratio of 10 to one case to minimize confounding factors. Most published studies in hospitalised patients were conducted in centres with long hospital stay [63–65]. In contrast, the hospitals where this study was done had a mean length of stay of 4.4 days. Hence, this population is different from most cohort of published results, which could contribute to the relatively lower fall rate seen.

We did not rely on incident reporting, instead actively enquired from the patient if they had fallen in the previous 24 h. Though this is not ideal, it could still reduce the reporting bias of obtaining fall experience solely from healthcare workers, and was less likely to miss any fall event. Most studies on falls have been community studies. Published data from hospital-based studies have been dependent on incident reporting or from a dedicated falls nurse. Our study was also able to explore the significance of previous falls as other studies had noted previous falls as a risk but did not test its relation to site of event [66–68].

We chose controls based on proximity to the faller. This could have inadvertently led to over-representation of certain diagnoses [69]. However, an advantage is that a case will likely have controls from similar ward (whether medical, surgical or orthopaedics, or first vs third class).

Type of diagnosis or comorbidities (e.g., frailty, impaired mental status, urinary incontinence) impaired mobility, type of surgical procedure and type of medication were noted in previous studies as risk factors for falls in older adults [44, 45, 59–62, 70]. Nonetheless, we could not analyse these known associated factors such as type of diagnoses, comorbidity, type and duration of medication, opting instead for counts for these variables, due to the varied responses and sample size. This could have confounded the final results, as the significant risk factors of falls identified this study might be the results of these confounders as older age is associated with increasing number and type of comorbidities [59]. For instance, risk of falls may also be influenced by the use of assistive device as they may be a surrogate for mobility or other diseases, such as frailty [59, 61, 62] that is associated with higher risk of falls, and when frailty is not investigated in a study, it could be a missed risk factor. During the study period, hospitals did not have ICD classification during the inpatient stay, nor did the facilities have electronic medical records. This, and insufficient numbers for these risk factors, affected the power of the analyses.

Furthermore, other factors that could influence falls that were not collected including nurse-to-patient ratio, incontinence, presence of indwelling catheter, urgency or need for frequent toileting. We could not account for confusion, a common phenomenon amongst admitted elderly [45] due to challenges to measure cognitive impairment by non-medical enumerators. As forementioned, we did not implement a frailty measure as the concept of frailty was relatively new in our setting during the study period and tools for frailty measure were not available during that time. Nonetheless, we had indirect measures of impaired mobility, such as the Barthel Index, and not by direct observation, performance or measured activity levels of subjects while in hospital. We did not explicitly exclude bed-bound patients, and surgical/orthopaedic wards might have lesser number of mobile subjects that in turn could account for lesser number of falls, although numbers from these wards were small (14 cases). The Barthel Index could not adequately capture these variations. Hence, future research on falls in general wards could consider incorporating these factors. Thus, there is a need to further define the profile of higher risk fallers in hospitals especially looking into risks that were not covered in this study as more detailed evidence could assist in targeted interventions .

Additionally, Hawthorne effect could be present and hospital personnel might have modified their behaviours. Enumerator turnover could have contributed to lower rates of falls in some hospitals or areas, as this led to lapses in the daily interviews with inpatients. Additionally, although we had substantial cohort involved, the 14,108 subjects from 12 hospitals of different types and the number of hospitals involved in the study was 10% of the total MOH hospitals in the nation, the study population differed from the MOH inpatient population, with less representation from minority groups. Hence, the results may not truly reflect the national picture even though we applied weights in the analysis. Lastly, as this study did not prospectively look at patients on fall outcomes from admission onwards, we are unable to infer causation, an area for future research.

## Conclusions

Falls in acute general wards were under reported and the method of active daily enquiry from patients on falls produces better detection than incident reporting. Risk factors identified in this study could complement existing strategies in identifying those at higher risk, and modification of extrinsic factors may help in reducing falls occurrence in non-intensive care areas of acute hospitals. Factors such as history of indoor or in-hospital fall, having four or more clinical diagnoses or exiting from weaker side and residence history may help to identify those at higher risk while addressing factors such as transfer bars, call bells, walking aids and light switches may help in reducing falls risk. It is hoped that the results of this study could guide fall prevention strategies for the older patients admitted to acute hospitals, and future empirical exploration could assess if implementing these measures lead to reduced events in different settings.

## Data Availability

All data generated or analysed during this study are available through the corresponding author upon request.

## Appendix

**Table 5:** Charges for general ward, number of beds and estimated number of admissions in MOH hospitals

Ward Class	Daily general ward charges (without air-conditioning) (MYR)	Daily general ward charges (with air-conditioning) (MYR)	Total number of beds for all types of ward in mid 2015	Estimated number of paid admissions to all types of ward in MOH hospitals in 2015
First Class Ward	﻿–	﻿–	2271	16,000
Single bed room	90	120	﻿–	﻿–
Double bed room	60	90	﻿–	﻿–
Room with three or more beds	45	60	﻿–	﻿–
Second Class Ward	25	40	3251	16,000
Third Class Ward	3	3	35,226	1,968,000
Total	﻿–	﻿–	40,748	2,000,000

## Abbreviations

IDS: Information and Documentation System
LOS: Length of stay
MOH: Ministry of Health
OR: Odds ratio
PMEM: Preliminary main effect model
SRS: Simple random sampling
